# Serum Neurofilament Light Chain and Glial Fibrillary Acidic Protein as Biomarkers in Hereditary Transthyretin Amyloidosis Polyneuropathy

**DOI:** 10.1111/jns.70104

**Published:** 2026-02-04

**Authors:** Valentin Loser, Pascal Benkert, Alex Vicino, Nicolas Ghika, Pansy Lim Dubois Ferrière, Chantal Daigneault, Thierry Kuntzer, Aleksandra Maleska Maceski, Jens Kuhle, Marie Théaudin

**Affiliations:** ^1^ Nerve‐Muscle Unit, Service of Neurology, Department of Clinical Neurosciences Lausanne University Hospital, University of Lausanne Lausanne Switzerland; ^2^ Multiple Sclerosis Centre and Research Centre for Clinical Neuroimmunology and Neuroscience (RC2NB), Departments of Biomedicine and Clinical Research, University Hospital and University of Basel Basel Switzerland

**Keywords:** amyloidosis, ATTR, GFAP, neurofilament, NfL, *Z*‐score

## Abstract

**Background and Aims:**

In individuals with hereditary transthyretin amyloidosis (ATTRv) polyneuropathy, monitoring of disease progression and treatment response is crucial. The objective is to determine if serum neurofilament light chain (sNfL) and serum glial fibrillary acidic protein (sGFAP) are reliable biomarkers of ATTRv polyneuropathy.

**Methods:**

We included 48 ATTRv individuals (38 symptomatic, 10 asymptomatic). Yearly assessments (over 4 years) included a full clinical examination with disease severity and functional scores, electrochemical skin conductance, nerve conduction studies, and measurement of sNfL and sGFAP levels. Using a reference database, sNfL and sGFAP were converted to *Z*‐scores (zNfL and zGFAP).

**Results:**

Median zNfL was −0.50 in asymptomatic, 1.44 in converters, and 2.46 in symptomatic subjects. zNfL > 1.42 discriminated symptomatic from asymptomatic subjects (AUC 0.936), not zGFAP (AUC 0.588). zNfL, not zGFAP, correlated with most clinical and electrophysiological neuropathy severity scales. Two asymptomatic carriers became symptomatic during follow‐up. In one of them, a significant rise in zNfL occurred 1 year before symptomatic transition.

**Interpretation:**

In ATTRv, zNfL correlates with neuropathy severity and symptomatic transition. A zNfL > 1.42 may discriminate symptomatic from asymptomatic subjects. zGFAP is not a reliable biomarker of polyneuropathy in ATTRv. Routine use of NfL should be based on deviation measure such as *Z*‐score.

**Abbreviations**
ATTRv: hereditary transthyretin amyloidosis
BMI: body mass index
CADT: Compound Autonomic Dysfunction Test
CMAP: compound muscle action potential
ESC: electrochemical skin conductance
FAP: familial amyloid polyneuropathy
GFAP: glial fibrillary acidic protein
HC: healthy controls
NCS: nerve conduction studies
NfL: neurofilament light chain
NIS: Neuropathy Impairment Score
NIS‐LL: NIS lower limb
NIS‐UL: NIS upper limb
PND: Polyneuropathy Disability Score
QOL‐DN: Norfolk Quality of Life in Diabetic Neuropathy
ROC: receiver operating characteristic
R‐ODS: Rasch‐built Overall Disability Score
SFN‐SIQ: Neuropathy‐Symptom Inventory Questionnaire
sGFAP: serum GFAP
SNAP: sensory nerve action potential
sNfL: serum NfL
TTR: transthyretin
zGFAP: GFAP *Z*‐score
zNfL: NfL *Z*‐score

## 1 Introduction

Hereditary transthyretin amyloidosis (ATTRv) is a rare autosomal dominant disease caused by a point mutation in the transthyretin (TTR) gene [1]. The pVal50Met mutation is the most frequently reported and is the cause of an endemic disease in northern Portugal, Sweden, and Japan. In Switzerland, most individuals are of Portuguese ancestry, and a few are late‐onset non‐endemic European individuals, whose clinical characteristics are similar to the French ATTRv subjects [2]. ATTRv presents as a life‐threatening multisystem disease, characterized by a disabling length‐dependent axonal polyneuropathy with prominent autonomic features [1]. Significant advances in disease modifying therapies have been achieved in the last decade, with treatment being able to dramatically suppress TTR production, thereby slowing or halting clinical progression of the disease [3, 4, 5, 6]. Early treatment initiation is recommended, as those treatments can delay disease progression, but do not allow a recovery of existing deficits [7]. Individuals with early disease may have no or only subtle neuropathy symptoms and signs, which cannot be easily quantified by current clinical or electrophysiological scales. As a result, there is a need for a sensitive and simple biomarker that could reliably identify disease onset and progression.

Neurofilament light chain (NfL) is a scaffolding protein exclusively expressed in neuronal cytoskeleton and is released into the extracellular space following neuro‐axonal injury. NfL levels in the serum (sNfL) have emerged as a potential biomarker of several neurological conditions [8]. In ATTRv amyloidosis, studies have indicated that sNfL may be useful to monitor disease progression, severity, and response to treatment [9, 10, 11, 12, 13, 14, 15, 16, 17, 18, 19, 20, 21, 22]. We have previously suggested, in a small cohort of 20 ATTRv individuals, the potential use of sNfL as a reliable biomarker of disease severity [14]. Few longitudinal assessments of sNfL have been performed to date, and appropriate cut‐off values for transition to symptomatic disease have yet to be established [18, 19]. Also, those studies used raw sNfL values, which are subject to biases depending largely on age (concentration increases with age) and to a lesser extent body mass index (BMI) (concentrations decrease with higher BMI). The use of age‐ and BMI‐corrected *Z*‐scores offers a more robust approach to sNfL analysis [23].

Glial fibrillary acidic protein (GFAP) is an intermediate filament of astrocytes and a biomarker of astrocytic damage and activation [24, 25]. Studies have recently suggested that blood GFAP could be a biomarker in some central nervous system disorders such as Alzheimer disease or multiple sclerosis [26, 27]. GFAP is also expressed in non‐myelinated Schwann cells to a minor extent [28]. As damage to unmyelinated small fibers occurs early in the course of the disease, GFAP could be a useful biomarker in ATTRv polyneuropathy [25]. Only three recent studies have investigated the role of serum GFAP (sGFAP) absolute concentration as a biomarker of ATTRv amyloidosis [22, 29, 30].

In this prospective study, we aimed to evaluate the use and longitudinal evolution of sNfL and sGFAP *Z*‐scores in a monocentric cohort of asymptomatic and symptomatic ATTRv subjects in a real‐life setting.

## 2 Material and Methods

### 2.1 Study Population

Subjects were prospectively recruited between October 2019 and February 2025 from the Lausanne University Hospital, a Swiss reference center for ATTRv amyloidosis. Inclusion criteria were: age ≥ 18‐year‐old, carrier of a pathogenic TTR mutation, symptomatic or not. Subjects with known acquired diseases or injuries of the CNS, including leptomeningeal amyloidosis or late‐onset central ATTRv symptoms, in which an increase in sNfL is to be expected, were excluded from the study. For longitudinal analysis, we defined three groups of subjects: subjects who remained asymptomatic during the whole follow‐up (asymptomatic, A), patients who were symptomatic at first evaluation (symptomatic, S), and subjects who converted from asymptomatic to symptomatic during the follow‐up (converters, C). Subjects were considered asymptomatic when they had either no clinical symptoms or mild subjective isolated sensory symptoms not suggestive of ATTRv and normal clinical examination, nerve conduction studies (NCS), and electrochemical skin conductance (ESC). Subjects were considered symptomatic when they had clinical symptoms suggestive of ATTRv polyneuropathy, an abnormal clinical examination, and abnormal NCS and/or ESC. Conversion was defined as the onset of symptoms suggestive of ATTRv polyneuropathy, in combination with clinical and electrophysiological (NCS or ESC) abnormalities. Converters and symptomatic subjects also showed evidence of amyloid deposition, either on tissue biopsy (skin, abdominal fat, or minor salivary glands) or on cardiac scintigraphy.

### 2.2 Clinical Assessment

Subjects were prospectively assessed at baseline and after 1 (T1), 2 (T2), 3 (T3), and 4 (T4) years. Clinical assessment at all time points included: demographics, BMI, full clinical examination with calculation of disease severity scores and functional scores, ESC assessment, and NCS. Sensory and autonomic subjective manifestations were rated with Small Fiber Neuropathy‐Symptom Inventory Questionnaire (SFN‐SIQ) and Compound Autonomic Dysfunction Test (CADT). Neuropathy‐related deficits were rated with Neuropathy Impairment Score (NIS) for both upper limbs (NIS‐UL) and lower limbs (NIS‐LL), and grip strength measured with Martin Vigorimeter in both hands. Grip strength was assessed three times in each hand, with the highest value retained for analysis. Functional outcome was assessed with the Polyneuropathy Disability Score (PND), familial amyloid polyneuropathy (FAP) stage, and Rasch‐built Overall Disability Score (R‐ODS). Quality of life was evaluated using the Norfolk Quality of Life in Diabetic Neuropathy (QOL‐DN).

### 2.3 Electrophysiologic Assessment

The NCSs were conducted using a Nicolet Viking EDX (Natus Medical GmbH). Standard techniques were used for percutaneous supramaximal stimulation and positioning of the surface electrodes in standardized conditions at a skin temperature of at least 33°C at the palm and 30°C at the external malleolus. Age‐ and height‐adjusted NCS reference values were used, according to the standards of our laboratory. Median, ulnar, fibular, tibial, and sural NCS were performed, and data from the right side of the body were analyzed. Results were translated into motor and sensory composite sum scores. The motor sum score is a composite of the negative amplitude of ulnar and fibular nerve compound muscle action potential (CMAP) in millivolt. The sensory sum score is a composite of the peak‐to‐peak amplitude of ulnar (orthodromic testing) and sural (antidromic testing) nerve sensory nerve action potential (SNAP) in microvolt. ESC was measured in hands and feet with Sudoscan.

### 2.4 Blood Sampling, sNfL and sGFAP Measurements and *Z*‐Score Transformation

All serum samples were frozen immediately and stored at −20°C. Samples were coded and sent blinded to the University Hospital of Basel, Switzerland, for analysis of sNfL and sGFAP levels. sNfL and sGFAP were measured using the Neurology 2‐plex B assay (Quanterix, USA) according to the manufacturer's instructions on the single molecule array (Simoa) HD‐X platform. We compared our subjects' sNfL levels with those of a reference database of 4532 healthy controls (HCs), aged 20–75 years [23], and our subjects' sGFAP levels with those of a reference database of 4297 HC, aged 20–75 years, to calculate sNfL (zNfL) and sGFAP *Z*‐scores (zGFAP) [31].

### 2.5 Ethical Approval

All procedures performed in this study were in accordance with the ethical standard of the institutional and national research committee as well as with the 1964 Helsinki Declaration and its later amendments. All subjects signed an informed consent form to participate in this prospective study, which was approved by the local institutional review board (CER‐VD 2019‐00301).

### 2.6 Statistics

*Z*‐score represents the number of SDs of our sample, compared to the mean of the HC group. A *Z*‐score of 0 is the mean value in the reference population accounting for those variables, a *Z*‐score of 1 means one standard deviation (SD) above the reference population value. For comparative analysis between the different groups (S, A, and C/pre‐ and post‐gene silencing treatment), NfL and GFAP values at different timepoints were pooled together. For descriptive statistics, mean, SD, median, and interquartile range (IQR) were used. For univariate comparisons of data, we used the Mann–Whitney *U* test for unpaired data and the Wilcoxon signed rank exact test for paired data. Receiver operating characteristic (ROC) curves were drawn by plotting the true‐positive fraction (sensitivity) against the false positive fraction (100%−specificity) for varying cut‐off values. Correlation between zNfL and zGFAP concentration and different variables were analyzed using Spearman correlation coefficient *ρ*. A Spearman correlation coefficient of < 0.2 was considered a very weak correlation, 0.20–0.39 a weak correlation, 0.4–0.59 a moderate correlation, 0.60–0.79 a strong correlation, and > 0.79 a very strong correlation [30]. Statistical significance was set at *p* < 0.05. Statistical analysis was performed using IBM SPSS Statistics for Windows, Version 30.0. Armonk, NY: IBM Corp.

## 3 Results

### 3.1 Baseline Characteristics of ATTRv Subjects

We included 48 genetically confirmed ATTRv subjects, 28 females and 20 males. Mean age at inclusion was 45.8 years (SD 13.6 years). Most subjects (*n* = 35, 73%) were of Portuguese ancestry, followed by subjects of Swiss (*n* = 7), Italian (*n* = 2), Scottish (*n* = 1), Ecuadorian (*n* = 1), Brazilian (*n* = 1), and Czech (*n* = 1) ancestry. The most frequent mutation was pVal50Met (*n* = 40, 83%). Other mutations were pGlu109Lys (*n* = 2), pPhe84Leu (*n* = 1), pVal142Ile (*n* = 1), pPhe84Ser (*n* = 1), pSer43Asn (*n* = 1), pGlu81Gly (*n* = 1), and pCys30Arg (*n* = 1). At baseline, there were 38 symptomatic patients and 10 asymptomatic carriers.

Among the 38 symptomatic individuals, 13 had undergone liver transplantation before inclusion. At baseline, 21 of them were not actively treated with anti‐amyloid therapy (including 8 liver transplanted patients). The 13 untreated non‐transplanted patients were diagnosed with ATTRv at baseline evaluation (hence the absence of treatment). They were all subsequently treated, before the one‐year follow‐up, either with anti‐amyloid therapy or liver transplantation (see below). The remaining 17 patients were either on patisiran (*n* = 13, included 3 liver transplanted patients) or tafamidis (*n* = 4). During follow‐up, among the 21 symptomatic untreated patients, 10 were started on patisiran, 2 on tafamidis, 1 on vutrisiran, 1 underwent a liver transplantation and 5 remained untreated (all were liver transplanted). Twelve patients switched treatment during follow‐up, four from tafamidis to patisiran (due to clinical progression in three cases) and eight from patisiran to vutrisiran (five for personal convenience and/or side effects and three for suspicion of clinical and/or biological progression/suboptimal biological response).

Symptomatic patients had a mild to moderate neuropathy, with a median PND score of 1 (range 1–4), a median FAP stage of 1 (range 1–3), a mean NIS score of 25.1 (SD 28.4, range 0–129.5), and a median R‐ODS score of 43.5 (range 16–48).

Demographics and clinical characteristics of symptomatic and asymptomatic subjects at baseline are summarized in Table 1.

**TABLE 1:** Clinical and demographic characteristics of symptomatic and asymptomatic ATTRv subjects at baseline.

	ATTR patients (*n* = 38)	Asymptomatic carriers (*n* = 10)	*p*
Age in years (mean, SD)	47.2 (13.7)	40.2 (12.3)	0.18
Female sex (%)	20 (53)	8 (80)	0.16
BMI in kg/m^2^ (mean, SD)	23.3 (4.6)	25.0 (5.3)	0.30
PND (median, IQR)	1 (1, 2)	0 (0, 1)	**< 0.001**
FAP (median, IQR)	1 (1, 1)	0 (0, 1)	**< 0.001**
SFN‐SIQ (median, IQR) (*n* = 47)	7 (6, 10)	3.5 (0.75, 5.25)	**< 0.001**
CADT (median, IQR) (*n* = 47)	12 (10, 15.5)	16 (13, 16)	**0.048**
RODS (median, IQR) (*n* = 47)	43.5 (36.5, 48)	48 (45.5, 48)	**0.010**
Norfolk QOL‐DN (median, IQR) (*n* = 46)	35.0 (14.5, 60.5)	14.0 (4.0, 15.5)	**0.002**
Total NIS UL (mean, SD)	7.5 (14.0)	0.1 (0.3)	**0.002**
Total NIS LL (mean, SD)	17.5 (17.3)	0.9 (1.5)	**< 0.001**
Total NIS (mean, SD)	25.1 (28.4)	1.0 (1.6)	**< 0.001**
Right handgrip with vigorimeter in kPA (mean, SD) (*n* = 46)	73.8 (21.4)	75.3 (14.9)	0.83
Left handgrip with vigorimeter in kPA (mean, SD) (*n* = 46)	70.6 (22.9)	67.1 (11.7)	0.59
ESC feet in uS (mean, SD) (*n* = 47)	38.5 (24.2)	76.3 (6.9)	**< 0.001**
ESC hands in uS (mean, SD) (*n* = 47)	48.2 (25.1)	65.7 (5.9)	**0.047**
NCS motor sum score in mV (mean, SD) (*n* = 47)	9.8 (5.8)	15.8 (3.1)	**< 0.001**
NCS sensory sum score in uV (mean, SD) (*n* = 47)	12.3 (14.5)	32.0 (17.2)	**< 0.001**

*Note:* Significant *p* values are in bold.

### 3.2 Longitudinal Course of ATTRv Subjects

Thirty‐eight subjects had a follow‐up at 1 year, 35 at 2 years, 29 at 3 years, and 21 at 4 years. During follow‐up, eight subjects remained asymptomatic (A), and two subjects converted from asymptomatic to symptomatic (C).

**FIGURE 1:**
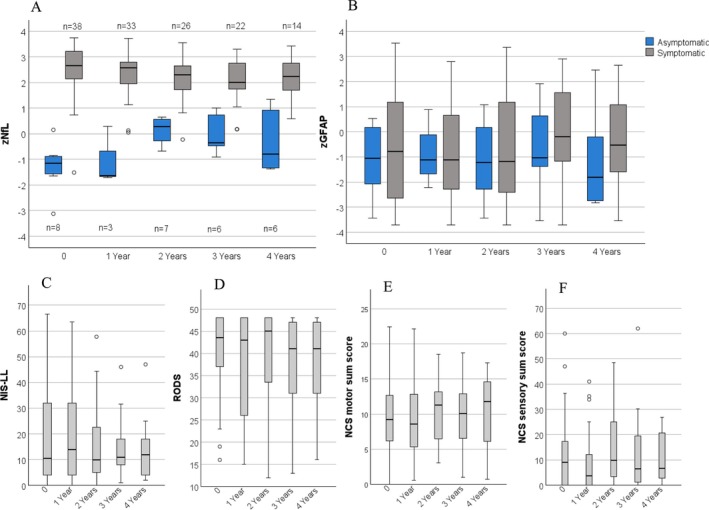
(A) In asymptomatic patients, zNfL slightly increased by 0.09/year. In symptomatic patients, zNfL slightly decreased by 0.1/year. (B) In asymptomatic patients and symptomatic patients, zGFAP values greatly varied between patients. There was no significant difference in NIS‐LL (C), RODS (D), NCS motor sum score (E), and NCS sensory sum score (F) in symptomatic patients during follow‐ups. The bold horizontal line represents the median, the top and bottom of the box the 25th and 75th quartile, respectively, and the extremity of the vertical lines the maximum and minimum values.

In Group A, median (IQR) zNfL was −1.15 (−1.60, −0.87) at baseline, −1.65 at T1, 0.28 (−0.44, 0.58) at T2, −0.35 (−0.58, 0.80) at T3 and −0.80 (−1.35, 1.02) at T4. zNfL remained < 1 in all carriers, except at the last follow‐up for one carrier, in which zNfL was 1.34 (Figure 1A). Median zNfL increased by 0.09/year. In Group S, median (IQR) zNfL was 2.65 (2.11, 3.23) at T0, 2.58 (1.93, 2.81) at T1, 2.30 (1.72, 2.76) at T2, 2.01 (1.74, 2.76) at T3 and 2.24 (1.64, 2.78) at T4. Median zNfL remained > 2 over follow‐up. Median zNfL decreased by 0.1/year (Figure 1A). In groups A and S, zGFAP values greatly varied between subjects, and over follow‐ups for each subject, sometimes differing by > 3 *Z*‐scores between two follow‐ups within the same subject (Figure 1B).

Symptomatic ATTRv patients remained clinically stable during follow‐up (Table S1 and Figure 1C–F) with stable NIS‐LL (*p* = 0.12) and RODS (*p* = 0.06). Only NCS sensory sum score (*p* = 0.53) slightly decreased during follow‐ups whereas NCS motor sum score slightly increased (*p* = 0.53). All symptomatic patients were treated by the first follow‐up, either with anti‐amyloid therapy or were previously liver transplanted. No symptomatic patients experienced significant worsening of neuropathy during the follow‐up.

In Group C, the first subject became symptomatic between T3 and T4 and was started on patisiran at this time. zNfL increased by 0.6/year before treatment introduction (Figure S1A). A significant rise in zNfL occurred at T3, that is, approximately 1 year before symptomatic transition, and a significant rise in zGFAP occurred at T2, that is, approximately 2 years before symptomatic transition. NfL and GFAP values after patisiran introduction are not available. In the second subject, the precise timing of symptomatic transition remained indeterminate. At T2, no sensory disturbances were reported in the lower limbs; however, a decrease in sural SNAP amplitude was noted relative to the previous evaluation. By T3, the subject reported mild sensory symptoms in the feet. Accordingly, it is presumed that the symptomatic transition occurred between T2 and T3. Vutrisiran was started at T3. zNfL was > 1 during the whole follow‐up. zNfL decreased by −0.05/year and zGFAP increased by 0.28/year before treatment introduction (Figure S1B).

### 3.3 Effect of Gene Silencing Therapy Introduction on NfL and GFAP

**FIGURE 2:**
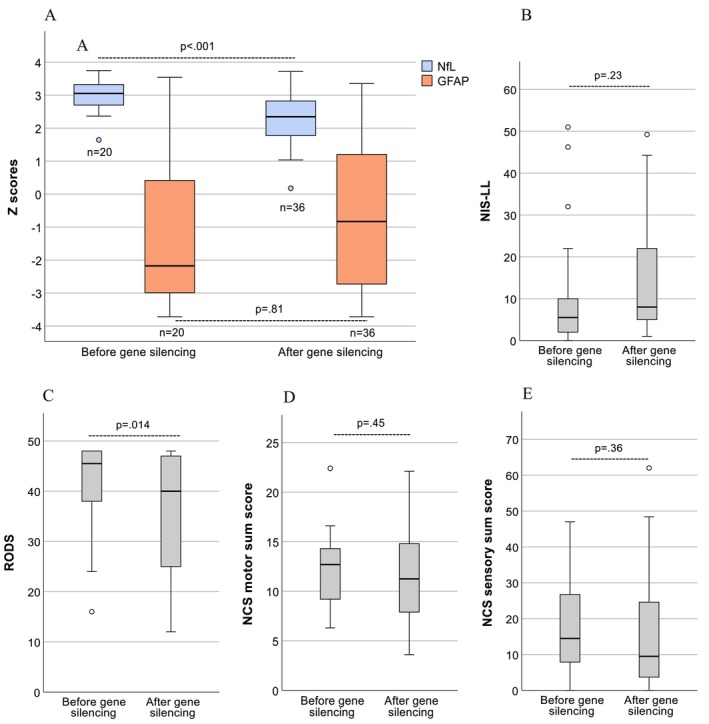
(A) zNfL decreased by 0.70 (*p* < 0.001) and zGFAP increased by 1.35 (*p* = 0.81) after gene silencing therapy introduction. There was no significant difference in NIS‐LL (B), NCS motor sum score (D), and NCS sensory sum score (E) in ATTRv patients before and after gene silencing therapy introduction. There was a significant decrease (*p* = 0.014) in RODS after gene silencing therapy introduction (C). The bold horizontal line represents the median, the top and bottom of the box the 25th and 75th quartile, respectively, and the extremity of the vertical lines the maximum and minimum values.

Gene silencing therapy was started during follow‐up in 15 symptomatic patients (14 patisiran and 1 vutrisiran) including 12 treatment‐naive patients, 2 patients who were previously liver transplanted, and 3 patients who clinically worsened on tafamidis treatment. In those 15 patients, median (IQR) zNfL was 3.05 (2.68, 3.34) before treatment introduction (*n* = 20 samples) and 2.35 (1.76, 2.84) after treatment introduction (*n* = 36 samples), which means a significant reduction in zNfL of 0.70 (*p* < 0.001) (Figure 2A). Median (IQR) zGFAP was −2.18 (−3.01, 0.43) before treatment introduction (*n* = 20 samples) and −0.83 (−2.83, 1.24) after treatment introduction (*n* = 36 samples), namely a non‐significant increase in zGFAP of 1.35 (*p* = 0.81). Among those 15 patients, there was a slight increase in NIS‐LL (*p* = 0.23), a slight decrease in NCS motor (*p* = 0.45), and NCS sensory (*p* = 0.36) sum scores, and a significant decrease in RODS (*p* = 0.014) after treatment introduction (Figure 2B–E). Gene silencing therapy introduction was associated with a rapid weight gain in 10/15 patients. In those 10 patients, mean weight gain was 5.7 kg, mean BMI gain was 1.83 kg/m^2^, and mean modified BMI (mBMI) gain (BMI in kg/m^2^ multiplied by serum albumin concentration in g/l) was 117.9.

### 3.4 Distribution of zNfL and zGFAP

**FIGURE 3:**
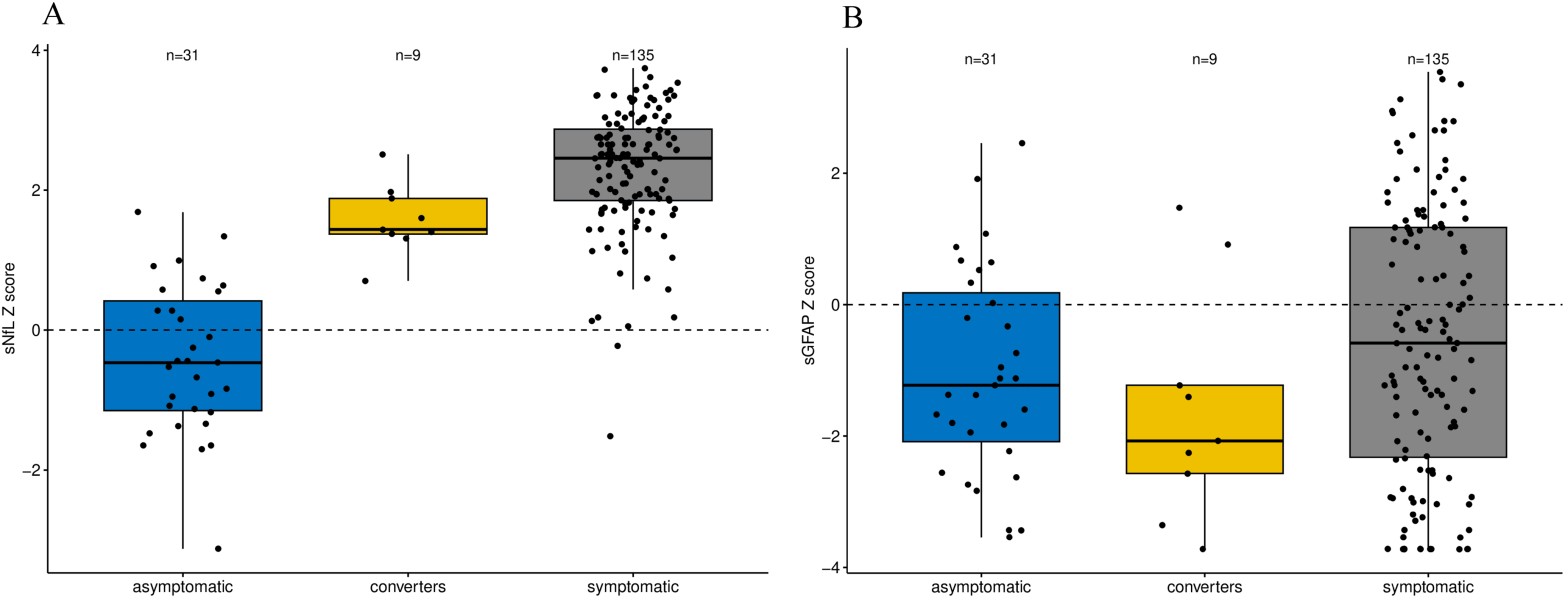
zNfL (A) and zGFAP (B) in asymptomatic, converters and symptomatic patients during the whole follow‐up. The bold horizontal line represents the median, the top and bottom of the box the 25th and 75th quartile respectively and the extremity of the vertical lines the maximum and minimum values. There is a significant difference in median zNfL between asymptomatic and converters (*p* = 0.002), between converters and asymptomatic (*p* < 0.001) and between symptomatic and asymptomatic patients (*p* < 0.001). There is no significant difference in median zGFAP between asymptomatic and converters (*p* = 0.241), between converters and symptomatic (*p* = 0.122) and between asymptomatic and symptomatic (*p* = 0.393).

**FIGURE 4:**
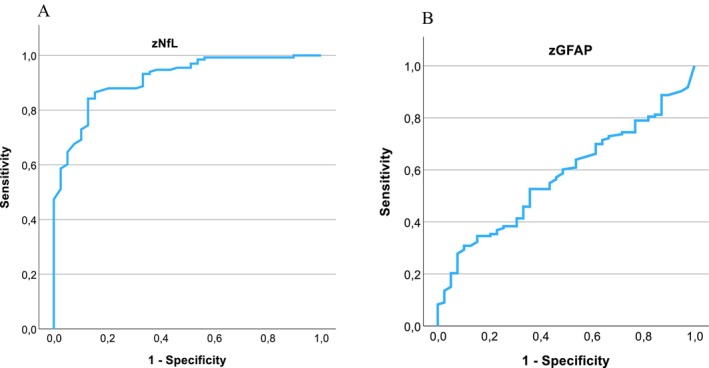
Area under the curve (AUC) of zNfL (A) to discriminate symptomatic from asymptomatic patients was 0.936 (*p* < 0.001). AUC of zGFAP (B) to discriminate symptomatic from asymptomatic patients was 0.588 (*p* = 0.115).

In Group A (*n* = 30 samples), median (IQR) zNfL was −0.50 (−1.22, 0.35), in Group C (*n* = 9 samples) 1.44 (1.34, 1.93) and in Group S (*n* = 133 samples) 2.46 (1.85, 2.87) (Figure 3A). ROC curve analysis was used to assess zNfL capacity to discriminate asymptomatic from symptomatic subjects. The area under the curve (AUC) to discriminate asymptomatic from symptomatic subjects was 0.936 (*p* < 0.001) (Figure 4A). zNfL of 1.42 discriminated these subjects with a sensitivity of 88.9% and a specificity of 91.9% (Youden index of 0.81).

In Group A, median (IQR) zGFAP was −1.18 (−2.31, 0.38), in Group C −2.08 (−2.96, −0.15) and in Group S −0.58 (−2.32, 1.18) (Figure 3B). sGFAP did not differ significantly between asymptomatic and converters (*p* = 0.241), converters and symptomatic (*p* = 0.122), and asymptomatic and symptomatic (*p* = 0.393). ROC curve analysis was used to assess zGFAP capacity to discriminate asymptomatic from symptomatic subjects. The AUC comparing asymptomatic to symptomatic subjects was 0.588 (*p* = 0.115) (Figure 4B).

### 3.5 Correlation of zNfL and zGFAP With Peripheral Neuropathy Severity

**FIGURE 5:**
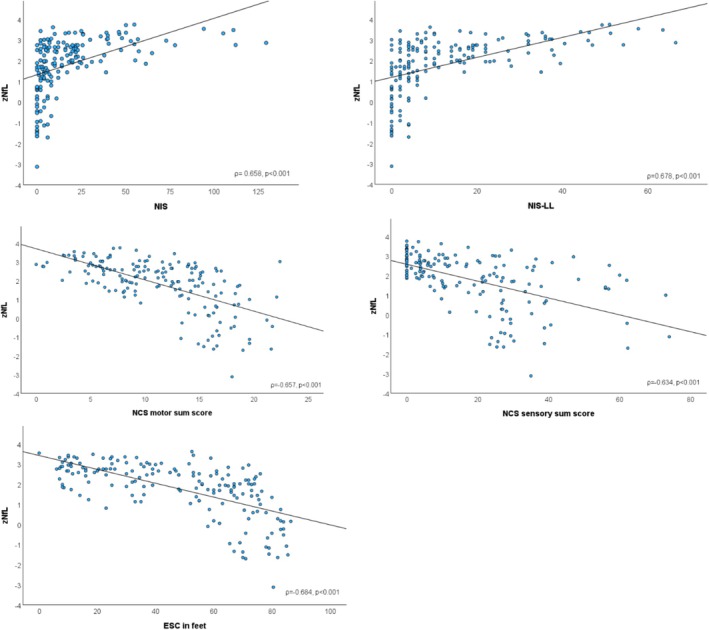
Correlation between zNfL and NIS (*ρ* = 0.658, *p* < 0.001); NIS‐LL (*ρ* = 0.678, *p* < 0.001); ESC in feet (*ρ* = −0.684, *p* < 0.001); NCS motor sum score (*ρ* = −0.657, *p* < 0.001), and NCS sensory sum scores (*ρ* = 0.634, *p* < 0.001).

Correlations between zNfL (*n* = 172 samples), zGFAP (*n* = 172 samples), and clinical and electrophysiological disease severity scores are displayed in Table S2. There was a strong correlation (*ρ* ≥ 0.6) between zNfL and NIS (*ρ* = 0.658, *p* < 0.001), NIS‐LL (*ρ* = 0.678, *p* < 0.001), ESC in feet (*ρ* = −0.684, *p* < 0.001) and NCS motor (*ρ* = −0.657, *p* < 0.001) and sensory (*ρ* = 0.634, *p* < 0.001) sum scores (Figure 5), and a moderate correlation (*ρ* = 0.4–0.6) with PND (*ρ* = 0.578, *p* < 0.001), FAP (*ρ* = 0.526, *p* < 0.001), Norfolk QOL‐DN (*ρ* = 0.565, *p* < 0.001), SFN‐SIQ (*ρ* = 0.569, *p* < 0.001), RODS (*ρ* = −0.501, *p* < 0.001), and ESC in hands (*ρ* = −0.521, *p* < 0.001).

There was neither strong nor moderate correlation between zGFAP and clinical and electrophysiological disease severity scores. Of note, the correlation between zNfL and zGFAP was weak (*ρ* = 0.184, *p* = 0.016).

## 4 Discussion

In this prospective monocentric study, we validate the use of sNfL *Z* score (zNfL) in a real‐life ATTRv population to monitor disease onset, disease severity and progression, and response to gene silencing treatment.

zNfL levels were elevated in patients with symptomatic ATTRv polyneuropathy compared to asymptomatic carriers, whereas asymptomatic carriers exhibited zNfL values comparable to those of HC (i.e., *Z*‐scores close to 0). zNfL was also significantly increased in converter subjects (median zNfL = 1.44) relative to asymptomatic carriers. A zNfL threshold of 1.42 effectively discriminated symptomatic from asymptomatic individuals, with a sensitivity of 88.9% and a specificity of 91.9%. Notably, this cut‐off value closely aligns with the threshold previously reported in our earlier study (1.45), thereby reinforcing its potential utility in routine clinical practice [14]. Pending further validation in larger cohorts, a zNfL cut‐off of 1.42 may be proposed as a supportive criterion of clinical symptomatic transition.

Absolute sNfL concentrations were significantly higher in converters compared to asymptomatic carriers, in symptomatic patients compared to converters, and in symptomatic patients compared to asymptomatic carriers. These findings are consistent with previous reports, where plasma/serum NfL levels were 3‐ to 10‐fold higher in symptomatic patients relative to asymptomatic carriers or HC [9, 10, 11, 12, 13, 14, 15, 16, 17, 18, 19, 20, 21, 22]. Across these studies, there was marked variability in reported mean/median sNfL values among ATTRv subjects, with highly inconsistent thresholds used to distinguish symptomatic from asymptomatic individuals, ranging from 10.6 to 64.5 pg/mL [10, 12, 14, 16, 17, 18, 22]. This heterogeneity likely reflects the influence of age and BMI on sNfL levels. Weight loss is a known marker of ATTRv polyneuropathy progression and is primarily attributed to autonomic dysfunction [32]. Conversely, gene‐silencing therapies are often associated with weight regain, as previously demonstrated in the APOLLO trials with patisiran [4, 32]. Given the inverse relationship between sNfL levels and BMI, weight fluctuations may impact sNfL concentrations. The use of *Z*‐scores helps to account for these variations, and our data show that the observed reduction in sNfL levels following initiation of gene‐silencing therapy is not solely attributable to weight gain, as zNfL values also decreased (see following section). This emphasizes the importance of using normalized metrics such as *Z*‐scores derived from age‐ and BMI‐adjusted reference datasets, instead of absolute concentrations. To our knowledge, application of NfL *Z*‐scores in ATTRv amyloidosis cohorts has not been reported previously, except in our earlier study, although this approach has been well established in multiple sclerosis and, more recently, in chronic inflammatory demyelinating polyradiculoneuropathy cohorts [23, 33, 34, 35, 36].

We also demonstrate that zNfL is well correlated with neuropathy clinical severity. zNfL correlates with most disease severity and functional scores, especially the NIS and NIS‐LL scales, and with electrophysiological markers of neuropathy, especially the NCS motor and sensory sum scores and ESC in feet. The correlation of serum or plasma NfL with disease severity scales [9, 10, 11, 12, 13, 14, 17, 18, 20, 21, 22] and NCS parameters [20] was already demonstrated previously. zNfL can hence be used as a biomarker of disease progression and severity.

During longitudinal follow‐up, zNfL levels remained stable in asymptomatic carriers with only a negligible increase over time. In all asymptomatic individuals, zNfL values remained below 1.0, except for a single timepoint in one subject. Due to the absence of subsequent follow‐up in this case, it remains unclear whether this subject eventually converted to the symptomatic stage. Previous data showed that sNfL levels remained stable in asymptomatic TTR mutation carriers [18, 19]. Our findings indicate that both zNfL and sNfL remain relatively stable in asymptomatic non‐converters, supporting a strong correlation with clinical stability.

In converters, zNfL increased at an average rate of 0.28 per year, while sNfL increased by 0.94 pg/mL/year. Berends et al. reported a higher rate of sNfL increase in converters (0.3 pg/mL/month) [19], though direct comparison is limited by the small number of converters in our cohort. In the first converter, a marked increase in both zNfL and sNfL was observed 1 year prior to symptomatic transition, with zNfL rising from 1.41 to 2.51 and sNfL from 12.0 to 23.1 pg/mL, exceeding the previously established zNfL cut‐off of 1.42. In the second converter, the transition occurred shortly after inclusion (approximately 1 year), precluding a detailed analysis of pre‐transition sNfL kinetics. Nonetheless, zNfL levels remained consistently elevated during follow‐up (1.60 at T0, 1.37 at T1, 1.98 at T2, and 1.44 at T3), exceeding the 1.42 threshold at 3 out of 4 timepoints. In line with our observations, Berends et al. also reported that increases in sNfL often preceded clinical symptom onset by several years in asymptomatic TTR carriers [19]. These findings suggest that a significant rise in sNfL and/or zNfL may precede symptomatic transition by a prolonged period, a hypothesis that warrants validation in larger prospective cohorts. Such early increases in sNfL/zNfL could represent a valuable biomarker for identifying asymptomatic ATTRv carriers at high risk of symptomatic conversion.

In symptomatic ATTRv patients receiving gene‐silencing therapy, we observed a slight decrease in zNfL levels over time, with an annual reduction of −0.1/year. This relative biological stability aligns with previous findings [18, 19]. The initiation of gene‐silencing therapy (either patisiran or vutrisiran) was associated with a reduction in zNfL (mean decrease of −0.70) in the subsequent years. These observations suggest that gene‐silencing therapies may effectively halt ongoing axonal injury and support the hypothesis that at least part of the neuronal damage in ATTRv amyloidosis may be reversible. Importantly, it remains unclear whether a suboptimal decrease—or even an increase—in zNfL or sNfL after therapy initiation could serve as an early biomarker of treatment resistance or predict future neuropathy progression. In our cohort, the reduction of sNfL levels was not immediately paralleled by clinical recovery, with even a slight functional worsening (decrease in RODS) after treatment initiation. This discrepancy may reflect a temporal dissociation between the reduction of axonal injury—reflected by decreased sNfL/zNfL levels, which can occur over weeks to months—and the slower processes of axonal regeneration and functional reinnervation, which may require several months to years to translate into clinical improvement. In addition, some patients may underestimate the functional impact of their neuropathy before diagnosis, whereas greater awareness and acceptance of the diagnosis may lead to lower RODS scores afterwards. Moreover, a delay of several months between diagnosis and initiation of anti‐amyloid therapy, due to insurance reasons in Switzerland, may account for true neurological worsening.

Concerning GFAP, we observed no significant differences in zGFAP levels between symptomatic patients, asymptomatic carriers, and converters, and no correlation between zGFAP and clinical disease severity, functional scales, or electrophysiological parameters. Thus, zGFAP does not appear to be associated with neuropathy severity or symptomatic transition. The diagnostic performance of sGFAP in discriminating between HC and ATTRv subjects has already been reported as poor by Agnello et al. (AUC 0.67) [30]. In our study, the diagnostic performance of zGFAP in discriminating between asymptomatic and symptomatic ATTRv subjects was similarly poor (AUC 0.588).

We also observed substantial variability in individual zGFAP values during follow‐up, both within the same subject and across different subjects. Moreover, zGFAP appeared to increase following the initiation of gene‐silencing therapy, in contrast to zNfL. An elevation of zGFAP prior to symptomatic transition was observed in only one of the two converters. These results contrast with three recently published studies, which reported higher serum GFAP levels in symptomatic ATTRv patients compared with HC [22, 29, 30]. In our cohort, median zGFAP values were negative across all three groups, indicating that median sGFAP concentrations were lower than those in HCs. It is noteworthy that two of the three previous studies reported conflicting findings: the first found similar sGFAP levels between asymptomatic carriers and HC [22], whereas the second reported higher sGFAP levels in asymptomatic carriers compared with controls [29]. In our study, we did not confirm zGFAP as a reliable biomarker for disease detection, disease severity, or symptomatic transition. This suggests that sGFAP alone may not represent a sufficiently sensitive biomarker for ATTRv diagnosis. Given that sGFAP is predominantly expressed in CNS astrocytes, its preferential localization in the central nervous system may explain the lack of association with peripheral nerve disorders. Furthermore, since sGFAP concentrations are influenced by age, BMI, and sex, the use of GFAP *Z*‐scores derived from age‐, BMI‐, and sex‐adjusted reference databases is warranted.

In conclusion, sNfL represents a promising biomarker of peripheral neuropathy in ATTRv amyloidosis. The use of absolute concentration values should be replaced by deviation measures, such as *Z*‐scores derived from age‐ and BMI‐adjusted HC cohorts. In this prospective study, we confirmed that zNfL can reliably discriminate asymptomatic from symptomatic subjects, with a cut‐off > 1.42 that could be proposed as a supportive criterion of symptomatic transition, and is strongly correlated with peripheral neuropathy severity. sNfL elevation likely precedes the clinical onset of neuropathy by several months/years, although larger‐scale studies are required to validate this observation. Serum GFAP, even when corrected using *Z*‐scores, did not prove to be a reliable biomarker of ATTRv peripheral neuropathy severity or symptomatic transition.

## Author Contributions

**V.L.:** conceptualization (supporting), formal analysis (lead), investigations (equal), methodology (equal), project administration (supporting), writing – original draft preparation (lead). **P.B.:** formal analysis (equal), investigations (supporting), writing – review and editing (equal). **A.V.:** writing – review and editing (equal). **P.L.D.F.:** data curation (equal), writing – review and editing (equal). **C.D.:** data curation (equal), writing – review and editing (equal). **T.K.:** writing – review and editing (equal). **A.M.M.:** formal analysis (equal), writing – review and editing (equal). **J.K.:** formal analysis (equal), writing – review and editing (equal). **M.T.:** conceptualization (lead), funding acquisition (lead), investigations (equal), methodology (equal), project administration (lead), resources (lead), writing – original draft preparation (supporting), writing – review and editing (equal).

## Funding

The authors have nothing to report.

## Disclosure

I hereby confirm that the manuscript complies with all instructions to authors; that authorship requirements have been met and the final manuscript was approved by all authors. I confirm that this manuscript has not been published elsewhere and is not under consideration by another journal. I confirm adherence to ethical guidelines.

## Ethics Statement

We confirm that we have read the Journal's position on issues involved in ethical publication and affirm that this report is consistent with those guidelines.

## Consent

All participants received full and clear information about the study and provided an informed consent to participate.

## Conflicts of Interest

Lausanne University Hospital received for Marie Théaudin honoraria for speaking, advisory boards, and compensation for congress participations from Alnylam, AstraZeneca, Sobi, outside the submitted work.

## Supporting information


**Table S1:** Clinical characteristics of symptomatic hATTR patients during follow‐ups. Significant *p* values are in bold.
**Table S2:** Correlation between severity and functional scales and electrodiagnostic parameters and zNfL and zGFAP. Significant *p* values are in bold.
**Figure S1:** Data in the two converters over follow‐up.

## Data Availability

The data that support the findings of this study are available from the corresponding author upon reasonable request.
