# Supplementary material for: Serum Neurofilament Light Chain and Glial Fibrillary Acidic Protein as Biomarkers in Hereditary Transthyretin Amyloidosis Polyneuropathy
Source: J Peripher Nerv Syst. 2026 Feb 4;31(1):e70104. doi: 10.1111/jns.70104 (PMC13285900; doi:10.1111/jns.70104)
Supplement: Supplementary file 1 — Table S1: Clinical characteristics of symptomatic hATTR patients during follow‐ups. Significant p values are in bold. Table S2: Correlation between severity and functional scales and electrodiagnostic parameters and zNfL and zGFAP. Significant p values are in bold. Figure S1: Data in the two converters over follow‐up. [file JNS-31-0-s001.docx]

**Supplementary materials**

**Table S1:** Clinical characteristics of symptomatic hATTR patients during follow-ups. Significant P-values are in bold.

|  | Baseline (n=38) | 1 year (n=33) | 2 years (n=26) | 3 years (n=22) | 4 years (n=14) |
| --- | --- | --- | --- | --- | --- |
| PND (median, IQR) | 1 (1,2) | 1 (1,2) | 1 (1,2) | 1 (1,2) | 1 (1,2) |
| FAP (median, IQR) | 1 (1,1) | 1 (1,1) | 1 (1,1) | 1 (1,1) | 1 (1,1) |
| SFN-SIQ (median, IQR) (n=47) | 7 (6,10) | 8 (6, 9.5) | 8 (6, 11) | 9 (6.5, 11) | 8.5 (4.8, 10.3) |
| CADT (median, IQR) (n=47) | 12 (10, 15.5) | 13 (10, 15.5) | 12 (11, 15) | 12 (9, 14) | 11.5 (8.8, 14) |
| RODS (median, IQR) (n=47) | 43.5 (36.5, 48) | 43 (25.5, 48) | 45 (32, 48) | 41 (30.3, 47.3) | 41 (29, 47) |
| Norfolk QOL-DN (median, IQR) (n=46) | 35.0 (14.5, 60.5) | 33.0 (14.0, 67.0) | 27.0 (6.0, 74.0) | 36.0 (5.8, 73.3) | 34.0 (9.2, 61.5) |
| NIS UL (mean, SD) | 7.5 (14.0) | 6.9 (13.2) | 5.5 (9.8) | 5.8 (8.4) | 4.5 (8.3) |
| NIS LL (mean, SD) | 17.5 (17.3) | 18.7 (17.5) | 15.2 (14.4) | 14.3 (10.9) | 13.5 (12.2) |
| NIS (mean, SD) | 25.1 (28.4) | 25.6 (27.7) | 20.8 (22.4) | 19.5 (18.3) | 18.0 (19.8) |
| Right handgrip with vigorimeter in kPA (mean, SD) (n=46) | 73.8 (21.4) | 73.1 (25.2) | 79.5 (24.3) | 72.6 (29.6) | 78.2 (27.9) |
| Left handgrip with vigorimeter in kPA (mean, SD) (n=46) | 70.6 (22.9) | 68.2 (24.7) | 75.2 (25.8) | 75.4 (30.7) | 78.4 (31.5) |
| ESC feet in uS (mean, SD) (n=47) | 38.5 (24.2) | 37.8 (22.9) | 39.7 (23.5) | 42.2 (21.9) | 39.4 (22.4) |
| ESC hands in uS (mean, SD) (n=47) | 48.2 (25.1) | 46.9 (21.4) | 49.7 (23.7) | 45.1 (23.6) | 42.1 (22.7) |
| NCS motor sum score in mV (mean, SD) (n=47) | 9.8 (5.8) | 9.5 (5.1) | 10.4 (4.2) | 9.8 (4.5) | 10.7 (4.7) |
| NCS sensory sum score in uV (mean, SD) (n=47) | 12.3 (14.5) | 9.6 (12.3) | 13.8 (13.7) | 12.3 (15.3) | 10.7 (10.1) |

**Table S2:** Correlation between severity and functional scales and electrodiagnostic parameters and zNfL and zGFAP. Significant p-values are in bold.

| Scale | zNfL | | zGFAP | |
| --- | --- | --- | --- | --- |
|  | Spearman correlation coefficient (ρ) | p-value | Spearman correlation coefficient (ρ) | p-value |
| PND | 0.578 | **<0.001** | 0.238 | **0.002** |
| FAP | 0.526 | **<0.001** | 0.209 | **0.006** |
| Norfolk QOL-DN | 0.565 | **<0.001** | 0.238 | **0.002** |
| SFN-SIQ | 0.569 | **<0.001** | 0.218 | **0.004** |
| CADT | -0.359 | **<0.001** | -0.248 | **0.001** |
| RODS | -0.501 | **<0.001** | -0.323 | **<0.001** |
| NIS | 0.658 | **<0.001** | 0.209 | **0.006** |
| NIS-UL | 0.390 | **<0.001** | 0.200 | **0.008** |
| NIS-LL | 0.678 | **<0.001** | 0.201 | **0.008** |
| Handgrip right | -0.191 | **0.013** | -0.124 | 0.108 |
| Handgrip left | -0.152 | **0.047** | -0.107 | 0.166 |
| ESC hands | -0.521 | **<0.001** | -0.273 | **<0.001** |
| ESC feet | -0.684 | **<0.001** | -0.205 | **0.007** |
| NCS motor sum score | -0.657 | **<0.001** | -0.133 | 0.088 |
| NCS sensory sum score | -0.634 | **<0.001** | -0.174 | **0.026** |
| sNfL |  |  | 0.184 | **0.016** |
| sGFAP | 0.184 | **0.016** |  |  |

**Figure S1:** data in the two converters over follow-up.

**
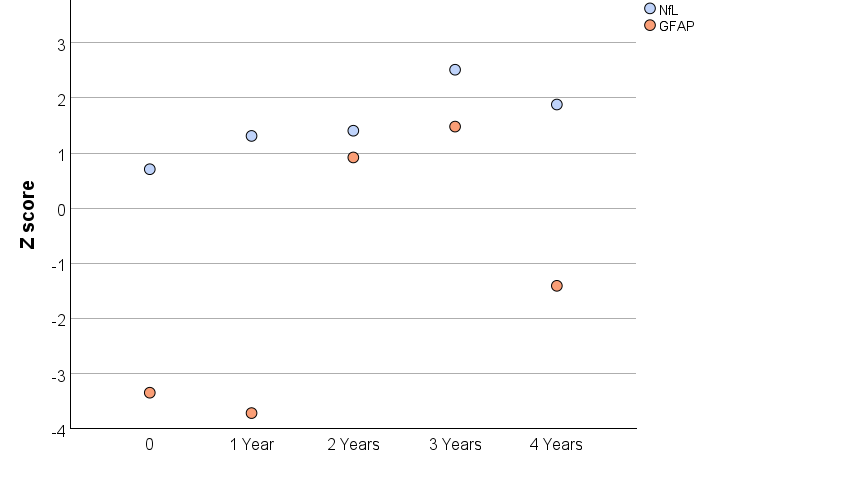
(A) Patient 1**

|  | 0 | 1 Year | 2 Years | 3 Years | 4 Years |
| --- | --- | --- | --- | --- | --- |
| zNfL | 0.71 | 1.31 | 1.41 | 2.51 | 1.88 |
| zGFAP | -3.35 | -3.72 | 0.92 | 1.48 | -1.41 |
| PND | 0 | 0 | 0 | 0 | 1 |
| NIS | 0 | 0 | 0 | 0 | 4 |
| ESC in uS (hands/feet) | 63/71 | 65/68 | 59/73 | 46/76 | 40/67 |
| NCS motor sum score in mV | 18.2 | 18.3 | 15.9 | 17.0 | 15.3 |
| NCS sensory sum score in uV | 39.3 | 56.9 | 56.0 | 57.1 | 27.1 |
| TTR silencer | None | | | | Patisiran |

Symptomatic transition

**(B) Patient 2**

**
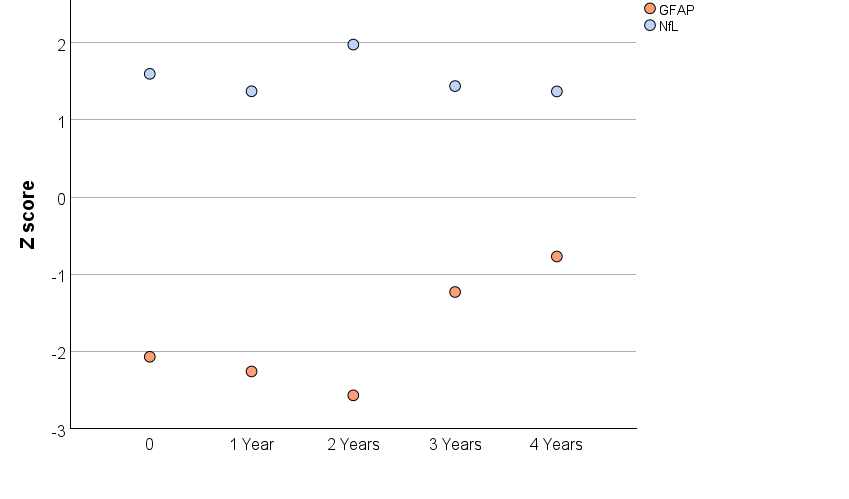
**

|  | 0 | 1 Year | 2 Years | 3 Years | 4 Years |
| --- | --- | --- | --- | --- | --- |
| zNfL | 1.60 | 1.37 | 1.98 | 1.44 | 1.37 |
| ZGFAP | -2.07 | -2.26 | -2.57 | -1.23 | -0.77 |
| PND | 0 | 0 | 1 | 1 | 1 |
| NIS | 3 | 4 | 5 | 5 | 11 |
| ESC in uS (hands/feet) | 57/70 | 63/54 | 61/56 | 68/75 | 61/78 |
| NCS motor sum score in mV | 8.4 | 8.0 | 8.3 | 6.7 | 7.4 |
| NCS sensory sum score in uV | 10.1 | 10.6 | 7.4 | 7.5 | 7.9 |
| TTR silencer | None | | | Vutrisiran | |

Presumed symptomatic transition
